# Different miRNA signatures of oral and pharyngeal squamous cell carcinomas: a prospective translational study

**DOI:** 10.1038/bjc.2011.29

**Published:** 2011-02-15

**Authors:** C B Lajer, F C Nielsen, L Friis-Hansen, B Norrild, R Borup, E Garnæs, M Rossing, L Specht, M H Therkildsen, B Nauntofte, S Dabelsteen, C von Buchwald

**Affiliations:** 1 Department of Oto-rhino-laryngology, Head and Neck Surgery, Rigshospitalet, University of Copenhagen, Blegdamsvej 9, Copenhagen 2100, Denmark; 2 Department of Clinical Biochemistry, Rigshospitalet, University of Copenhagen, Blegdamsvej 9, Copenhagen 2100, Denmark; 3 Department of Cellular and Molecular Medicine, University of Copenhagen, Blegdamsvej 9, Copenhagen 2100, Denmark; 4 Department of Oncology, Rigshospitalet, University of Copenhagen, Blegdamsvej 9, Copenhagen 2100, Denmark; 5 Department of Pathology, Rigshospitalet, University of Copenhagen, Blegdamsvej 9, Copenhagen 2100, Denmark; 6 Department of Oral Pathology and Medicine, University of Copenhagen, Blegdamsvej 3B, Copenhagen N 2200, Denmark

**Keywords:** oral squamous cell carcinoma, head and neck cancer, HPV, miRNA

## Abstract

**Background:**

MicroRNAs (miRNAs) are small non-coding RNAs, which regulate mRNA translation/decay, and may serve as biomarkers. We characterised the expression of miRNAs in clinically sampled oral and pharyngeal squamous cell carcinoma (OSCC and PSCC) and described the influence of human papilloma virus (HPV).

**Methods:**

Biopsies obtained from 51 patients with OSCC/PSCC and 40 control patients were used for microarray analysis. The results were correlated to clinical data and HPV status. Supervised learning by support vector machines was employed to generate a diagnostic miRNA signature.

**Results:**

One hundred and fourteen miRNAs were differentially expressed between OSCC and normal oral epithelium, with the downregulation of miR-375 and upregulation of miR-31 as the most significant aberrations. Pharyngeal squamous cell carcinoma exhibited 38 differentially expressed miRNAs compared with normal pharyngeal epithelium. Differences in the miRNA expression pattern of both normal epithelium and SCC were observed between the oral cavity compared with the pharynx. Human papilloma virus infection revealed perturbations of 21 miRNAs, most significantly in miR-127-3p and miR363. A molecular classifier including 61 miRNAs was generated for OSCC with an accuracy of 93%.

**Conclusion:**

MicroRNAs may serve as useful biomarkers in OSCC and PSCC. The influence of HPV on miRNA may provide a mechanism for the distinct clinical behaviour of HPV-infected tumours.

Head and neck cancer is the fourth most common cancer among men in The European Union (Black *et al*, 1997) and more than 21 000 patients die from cancer in the oral cavity and pharynx in the European Union each year (Bosetti *et al*, 2008). Squamous cell carcinoma of the head and neck (HNSCC) is a heterogeneous disease involving multiple anatomical localisations. Alcohol and tobacco are predisposing factors for developing HNSCC, but human papilloma virus (HPV) infection is also known to be associated with HNSCC, especially squamous cell carcinoma (SCC) of the oropharynx (tonsils). Human papilloma virus-positive tumours have distinct clinical and prognostic features, and discussions concerning whether or not patients with HPV-positive tumours should be regarded as a separate group and receive different treatment compared with HPV-negative tumours are ongoing. Finally, problems still remain in identifying HPV and showing the true effect of HPV in the complex environment of other competing oncogenic factors such as alcohol and tobacco.

In general, about half of the patients with HNSCC are cured. The clinical features as well as prognosis and treatment vary not only with HPV status, but also with different anatomical localisations and stage. For oral cavity SCC (OSCC), treatment comprises excision of the primary tumour followed by sentinel node biopsy or selective neck dissection (Bilde *et al*, 2006). Post-operative radiotherapy is indicated for advanced stages or in the case of a non-radical excision with regard to primary tumour or extra nodal cancer growth of the regional lymph nodes. For pharyngeal (PSCC) and laryngeal SCC, treatment is primarily radiotherapy with and without concomitant chemotherapy. All treatment modalities are accompanied by considerable side effects, which may have a major impact on the quality of life in these patients. In addition, even patients with the same anatomical localisation and stage may experience different outcome and treatment morbidity on the same treatment modality. Despite the development of advanced multidisciplinary treatment, survival has only increased modestly. For the development of new molecular targeted strategies and to identify patients who are more or less likely to respond to a given therapy, it is important to look into cancer biology and discover the molecular differences between the anatomical localisations as well as separating the viral from the smoking and alcohol-induced cancers.

MicroRNAs (miRNAs) are small 21–22*n* non-protein-coding RNAs that regulate mRNA translation and decay. It has become evident that miRNA plays a significant role in the development of human cancer, and the miRNA expression pattern is altered in many types of cancer. Some miRNAs have been characterised as tumour suppressors (Kozaki *et al*, 2008) and others as oncogenic (onco-miRs) (Iorio *et al*, 2005). MicroRNA patterns are tissue specific, and in addition, it is often possible to distinguish carcinomas from normal cells (Visone *et al*, 2007). MicroRNA expression profiling for head and neck cancers has been carried out in different studies, but mostly in cancer cell lines (Tran *et al*, 2007; Kozaki *et al*, 2008) or in a few solid tumours (Chang *et al*, 2008; Wong *et al*, 2008). Cell lines may not reflect the miRNA profiles of solid tumours because the particular culture conditions and clonal selection may radically change miRNA expressions. In most studies including larger patient materials, only a few miRNAs have been selected for further investigation (Childs *et al*, 2009). Furthermore, there have been little consistency in the published profiles, and the ongoing discovery of new miRNAs implies the need for new profiles in the search of relevant diagnostic as well as prognostic biomarkers. A number of miRNAs have been shown to be upregulated, whereas others were reduced. MicroRNA-21, which is overexpressed in a number of solid malignant tumours, was also found in the neoplastic head and neck cells and has been shown to be a prognostic marker in head and neck cancer (Childs *et al*, 2009). The role of HPV on miRNA expression in head and neck cancers is largely unknown.

In a translational perspective, we examined the global miRNA expression in a series of consecutive tumours or biopsies obtained from patients with OSCC, PSCC and controls, respectively. We report remarkable tissue specificity in both normal and malignant tissue and that HPV status affects the miRNA expression pattern. On the basis of these observations, we generated an miRNA-based classifier that could differentiate between normal and malignant tissue in the oral cavity. We conclude that progression from normal epithelium to SCC is followed by changes in miRNA expression and that miRNA-based signatures may in the future support diagnosis in head and neck cancer.

## Materials And Methods

### Patient characteristics

The Regional Scientific Ethical Committee (H-C-2008-080) approved the study. All patients gave informed consent for the use of tissue harvested at surgery.

In total, 100 patients had biopsies taken for RNA extraction. Twelve patients were excluded because the tumour cell content was low or the subsequent histological examination revealed another diagnosis than SCC. Eighty-eight cancer and control patients were eligible for analysis. Thirty patients with primary OSCC (stages I–IV) and 19 patients with primary SCC of oro- and hypopharynx (stages I–IV) were included. All cancer patients had histological documented SCC and the tumour grade of differentiation was evaluated according to the WHO criteria by an experienced pathologist (Eds Barnes, 2005). Tumour stage was evaluated by the primary study investigator by clinical examination and CT of the head and neck according to the TNM classification of Malignant Tumours from International Union Against Cancer (UICC) (Patel and Shah, 2005). Thirty-nine patients with non-neoplastic diseases within the head and neck region were used as controls, of whom 22 had biopsies taken from the pharynx and 17 had biopsies taken from oral cavity. Human papilloma virus status was examined for all patients by three modalities: PCR, p16^INK^ immunohistochemistry and *in situ* hybridisation (ISH).

### Tissue collection

Biopsies were harvested during primary surgery and placed directly in ‘RNA later’. The control group consisted of patients who obtained surgery for non-neoplastic diseases of the head and neck in the Department of Otolaryngology, Head and Neck Surgery, Rigshospitalet, and they were chosen to match the cancer patient group with regard to age, smoking status and biopsy site.

Control tissue from the tonsils was collected after surgery by carefully scraping the epithelium from the lymphoid tissue to avoid prominent infiltration of lymphoid tissue from the samples. Biopsies were frozen to −80°C within 10 min.

### Microarray analysis

#### RNA extraction

Total RNA was extracted and isolated by homogenising the frozen samples in Trizol (Invitrogen Corporation, Carlsbad, CA, USA) using a homogeniser (Tissuelyser; Qiagen GmbH, BY, Hilden, Germany). Briefly, total RNA was extracted from tissue lysate by phase separation with BCP (1-bromo-3-chloropropane) and RNA precipitation with isopropanol. After washing with alcohol, the RNA was eluted in RNAse free water. Total RNA concentration of samples was measured using a Nanodrop ND-1000 Spectrophotometer (NanoDrop Technologies Inc., Wilmington DE, USA). For microarray analysis, 1000 ng of total RNA was labelled with FlashTag Biotin RNA Labelling Kits from Genisphere and hybridised to Affymetrix GeneChip miRNA array according to the manufacturer's instructions.

#### Microarray

Affymetrix miRNA array chips identify miRNAs in 71 organisms, including small nucleolar RNAs and small Cajal body-specific RNAs in human beings. Content is derived from Sanger miRbase miRNA database V11. It is a one-colour array and includes 847 human miRNAs. Four copies of each miRNA probe are distributed on the array. To minimise batch variation, each batch was run with equal numbers of OSCC, oral controls, PSCC and pharyngeal controls. Twenty samples were labelled and hybridised in each batch. Raw data files were imported into Affymetrix miRNA QCTool and normalised using the quantiles normalisation and median Polish summarisation following a background correction that corrects for the GC content of the each particular probe.

#### mRNA expression arrays

RNA was amplified and labelled using the Ambion WT Expression Kit (Applied Biosystems, Life Technologies Corporation, Carlsbad, CA, USA) according to the manufacturer's instructions. The labelled samples were hybridised to Human Gene 1.0 ST GeneChip array (Affymetrix, Santa Clara, CA, USA). The arrays were washed and stained with phycoerythrin-conjugated streptavidin using the Affymetrix Fluidics Station 450, and the arrays were scanned in the Affymetrix GeneArray 2500 scanner to generate fluorescent images, as described in the Affymetrix GeneChip protocol. Cell intensity files were generated in the GeneChip Command Console Software (AGCC) (Affymetrix).

#### DNA extraction

DNA was extracted from the interphase of the Trizol and precipitated with ethanol. It was dissolved in lysate buffer and proteinase K, and after another precipitation with ethanol, the solutions were transferred to spin columns (NucleoSpin Tissue). After washing, DNA was eluted with water of 70°C.

Quantitative polymerase chain reaction (qPCR) validation was carried out on miRNAs selected from the miRNA microarray analysis in 20 samples. cDNA was prepared from 25 ng total RNA using TaqMan MicroRNA Reverse Transcription Kit and TaqMan MicroRNA Assays containing pre-designed primers for miR-125b, miR-187, miR-31, miR-181b and miR-375. hsa-miR-191 was used for endogenous control. Quantitative reverse transcription–PCR (QRT–PCR) reaction was performed using TaqMan Universal PCR Master Mix No AmpEras UNG, according to the manufacturer's instructions, all from Applied Biosystems. Each amplification reaction was performed in triplicate, and the median value of the three-cycle threshold was used for further analysis. For calculations of fold changes, we used the 2^−ΔCt^ method (Schmittgen and Livak, 2008).

#### Human papilloma virus

Polymerase chain reaction for HPV was performed using general primers MY09 and MY11 and GP5+/GP6+ (Hsing *et al*, 1996; Jacobs *et al*, 1997). Validation of the DNA quality and the efficacy of the PCR were carried out with PCR for the housekeeping gene *GAPDH*.

Immunohistochemistry for p16^INK4A^ was performed on formalin-fixed paraffin-embedded tissue slides pre-treated in DAKO PT Link, incubated with P16 (JC8) antibody, Santa Cruz (Santa Cruz Biiotechnology Inc., Santa Cruz, CA, USA), and visualised using DAKO Envision Flex+ system. Slides were evaluated by a pathologist as positive, if there was a strong diffuse staining of more than 50% of the tumour cells.

*In situ* hybridisation was performed on formalin-fixed paraffin-embedded, 4-*μ*m tissue sections using PathoGene HPV Screening Probe (Enzo Life Sciences, Lausen, Switzerland) in accordance with the manufacturer's instructions. The probe used was a ready to use cocktail of biotin-labelled HPV6, HPV11, HPV16, HPV18, HPV31, HPV33 and HPV51.

Positive staining was identified as red nuclear dots. Any definitive nuclear staining in the tumour cells was considered positive.

### Statistics

Log 2 intensities of the 847 human miRNAs were imported into the Data Analysis software package Qlucore Omnics Explorer 2.1. Class comparison analysis was performed using Student's *t*-test. Oral cavity and pharynx samples were analysed separately. For each tissue type, a comparison of cancers *vs* controls was performed, and an miRNA was defined as being differentially expressed between the groups if the *P*-value was below 0.01 and the fold change above 1.5. In the PSCC subgroup, we compared HPV-positive *vs* -negative cases, and in the OSCC group, we compared patient samples with and without lymph node metastases. In these analyses, an miRNA was defined as being differentially expressed between the groups if the *P*-value was below 0.02 and the fold change above 1.5.

### Visualisation of differentially expressed miRNAs

The built-in principal component analysis (PCA) tool in Qlucore Omnics Explorer was used only for visualisation of the grouping of samples using the genes selected in the class comparison. Hierarchical clustering was used for the visualisation of selected differentially expressed miRNAs using average linkage and Euclidean distance. Hierarchical clustering visualisation of both samples and variables is carried out using the Euclidean metric and data, where each variable has been normalised to mean 0 and variance 1.

### Formulation of classifier

The diagnostic classifiers were developed in ‘R’ v. 2.7. For all classification problems, the training of the classifiers inside the leave-one-out (LOO) loop consists of two steps: a univariate probe ranking and selection step, followed by fitting a support vector machine (SVM) on the sample division using the selected probes as covariates. All models were optimised by a grid search of *P*-value cutoffs (0.01, 5E-02, 1E-02, 5E-03, 1E-03, 5E-04,1E-04) and the cutoff resulting in a gene signature of optimal performance was used in the final model. The gene signature in the classifier was selected with Student's *t*-test with a *P*-value below 5E-04. Model fitting was carried out by training an SVM with a Gaussian kernel (Vapnik, 1999).

### Test set of classifier

To validate that the classifier can distinguish between OSCC and normal oral mucosa outside the training setting, a collection of biopsies from seven patients with OSCC and seven control patients were harvested independently of the original 48 oral patients in the study. All control biopsies were taken from the gingival mucosa from patients operated with tooth extractions at the Department of Odontology, Copenhagen University. RNA was extracted, labelled and hybridised to Affymetrix GeneChip miRNA Array with the same procedure as used for the original samples. Raw data files from the test set were imported together with the original training data set into Affymetrix miRNA QCTool and normalised. The 14 samples were used as an independent test set; the classifier was applied using the signature obtained from the original data set of 48 samples with the criterions described in the classifier.

### miRNA target prediction and gene ontology analysis

To predict mRNA changes, based on the observed miRNA profile, we implemented the method developed by Gallagher *et al* (2010). A gene list of predicted mRNA targets for the most significant aberrations in miRNA expression (more than twofold up or down between the normal and OSCC) was obtained using miR database miRDB. Only genes with a target score of more than 80 were considered and a prediction of which genes should be altered in view of multiple changes in miRNA concentration were made according to the method described by Gallagher *et al* (2010). This method provides a weighted miRNA inhibitor score (sum of effects) to the genes predicted as being targets of the regulated miRs. The overlap between the predicted target genes and the genes detected as present in the mRNA expression analysis was examined further. ‘Present genes’ was defined by having an average intensity above 100, when examining the gene expression arrays (HuGene ST 1.0; Affymetrix) of the OSCC and controls.

The effect of the up- and downregulated miRNAs was calculated separately as described by Gallagher *et al* (2010); however, we combined the two lists into one by adding the inhibitory effect of the upregulated miRNAs with the stabilising effect of the downregulated miRNAs. We used a cutoff on our weighted inhibitor score list of ±2, resulting in 497 genes that were used for the biological function and pathway analyses in Ingenuity system software.

To assign function to the genes with the highest and lowest miRNA inhibitor score, biological function analysis was performed using the Ingenuity system software. This analysis estimates whether a biological function or pathway is enriched in the gene list provided compared with a Reference Set of molecules (the Human Gene 1.0 ST GeneChip array platform (Affymetrix) using a right-tailed Fisher's exact test. It gives a significance value as a measure of the likelihood that the association between a set of genes in our experiment and a given process or pathway was due to random chance. A Benjamin–Hochberg (FDR) corrected *P*-value of less than 0.05 was regarded as statistically significant.

## Results

### Patients and tumours

Patient characteristics are summarised in Table 1. The median age of all patients was 60 years and the age distribution was very similar in the OSCC, PSCC and oral cavity control groups, but slightly lower in the pharyngeal control group. There were a higher proportion of women in the control groups compared with the cancer groups, whereas the distribution of smokers *vs* non-smokers was similar among the groups. Patients’ tumour and controls’ biopsy characteristics are listed in Table 2. There were a higher proportion of higher stages (stage III and IV) in the PSCC patients than in the OSCC patients, where most patients had stages I and II. The presence of HPV in the tumours was determined by PCR and this revealed only one HPV-positive control and 11 positive cancers among 90 samples. Two of the latter were excluded because the number of tumour cells in two samples was low. P16^INK4A^ immunostaining revealed 15 were HPV positive and by ISH seven were HPV positive. For ensuring to find the ‘true’ HPV caused and clinically relevant HPV-positive patient samples, all three tests (P16^INK4A^ immune, ISH and HPV DNA PCR) for HPV were carried out. Only patients who were tested positive for at least two of the three parameters were considered HPV positive. Only eight PSCC and one OSCC fulfilled this criterion and could be used to evaluate the significance of HPV.

### miRNA expression in oral and pharyngeal cancers

**Figure 1:**
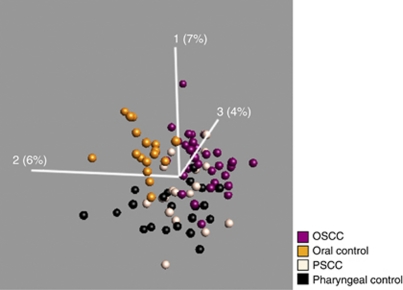
Principal component analysis of all four patient groups using all 847 miRs measured on the microarray. Oral squamous cell carcinoma (purple), oral cavity controls (gold), PSCC (off-white) and pharyngeal controls (black). The oral cavity controls and the OSCC samples cluster into two distinct groups. The PSCC samples showed a higher degree of heterogeneity and shows partial overlap with both the OSCC and the pharyngeal controls. The PCA analysis and visualisation was performed using the Qlucore Gene Expression Explorer (www.qlucore.com).

To obtain an overview of the differences and similarities of the miRNA expression among the different cancers and normal tissues groups, we first visualised the miRNA expression of all samples in a PCA including all variables. In the uncensored (using all miRs) PCA plot, the oral control samples and the OSCC samples clustered into two distinct groups (Figure 1). Furthermore, the PSCC samples showed a higher degree of heterogeneity and showed partial overlap with both the OSCC, as expected, and the pharyngeal controls. This may be due to a higher level of normal tissue cells in the PSCC biopsies. Overall, the PCA plot showed that miRNAs are able to separate normal oral mucosa from the tumours as well as separate normal oral from normal pharyngeal mucosa.

**Figure 2:**
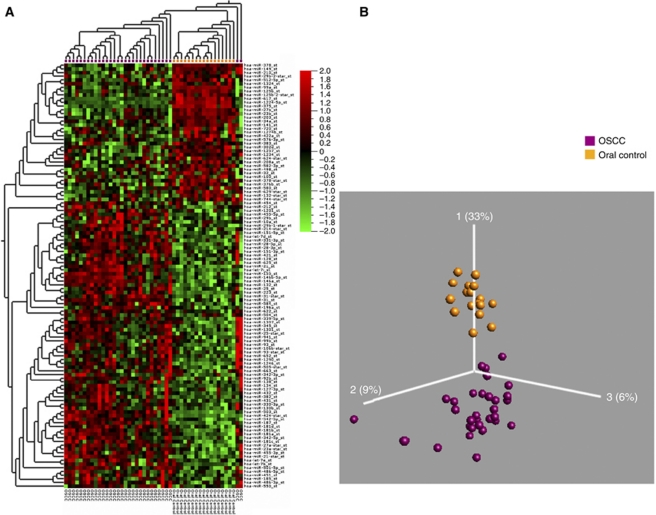
Visualisation of 114 differentially expressed miRs in OSCC and oral cavity controls (*P*<0.01, *q*<0.06) p. (**A**) Heatmap: Two distinct groups were observed. OSCC (purple) and oral cavity controls (gold). (**B**) PCA plot: OSCC (purple) *vs* oral cavity controls (gold). The heatmap and PCA visualisation was performed using the Qlucore Gene Expression Explorer (www.qlucore.com).

**Figure 3:**
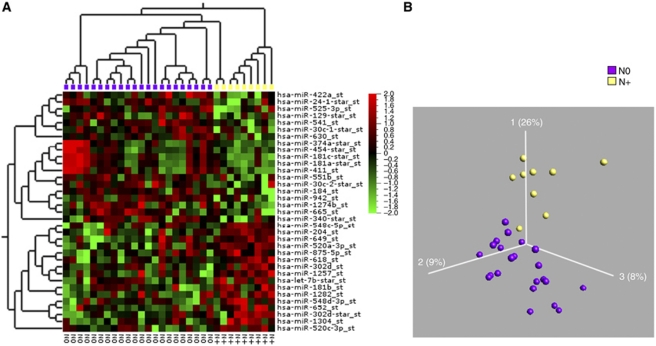
Visualisation of 18 differentially expressed miRs between the OSCC samples with lymph node metastases (yellow) and OSCC samples without lymph node metastasis (purple). *P*<0.02, *q*<0.6. (**A**) Heat map and (**B**) PCA plot.

We subsequently examined oral and pharyngeal tumour samples, respectively, with the corresponding normal biopsies in a censored class comparison analysis. Employing a significance levels of *P*<0.01 and *q*<0.06, 114 miRNAs were found to be differentially expressed between OSCC and oral cavity control samples (Figure 2). Thirty-six miRNAs were downregulated and 78 upregulated. The most significant aberration was a >18-fold downregulation of miR-375 in oral cancers. The highest upregulated miRNA was miR-31, which was increased 6.9-fold. The 25 most highly differentially expressed miRNAs are listed in Table 3. As miRNAs could be implicated in tumour progression and metastasis, we also compared miRNAs in OSCC from different stages and with or without lymph node metastasis. Tumours with different staging did not reveal major differences in the miRNA expression, so we subsequently grouped all tumours with metastasis and analysed them against the remaining tumours. Eighteen miRNAs were differentially expressed between OSCC with lymph node metastases and OSCC without lymph node metastases at a significance level of *P*<0.02 and a false discovery rate (FDR) of *q*<0.6 (Figure 3). However, the fold changes were small and ranged from −3 to 1.5 and none of the identified miRNAs were unique to any of the categories.

**Figure 4:**
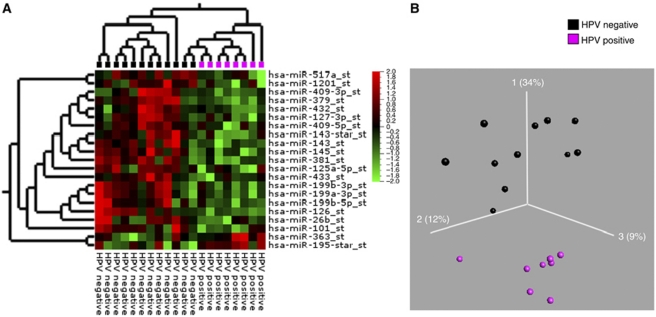
Visualisation of differentially expressed miRs in HPV-positive compared with HPV-negative PSCC. (**A**) Heat map: HPV-positive PSCC (purple) and HPV-negative PSCC (black). (**B**) PCA plot: HPV-positive PSCC (purple) *vs* HPV-negative PSCC (black). The miRs were selected with Student's *t*-test with *P*<0.02, *q*<0.36 and a fold change >1.5.

**Figure 5:**
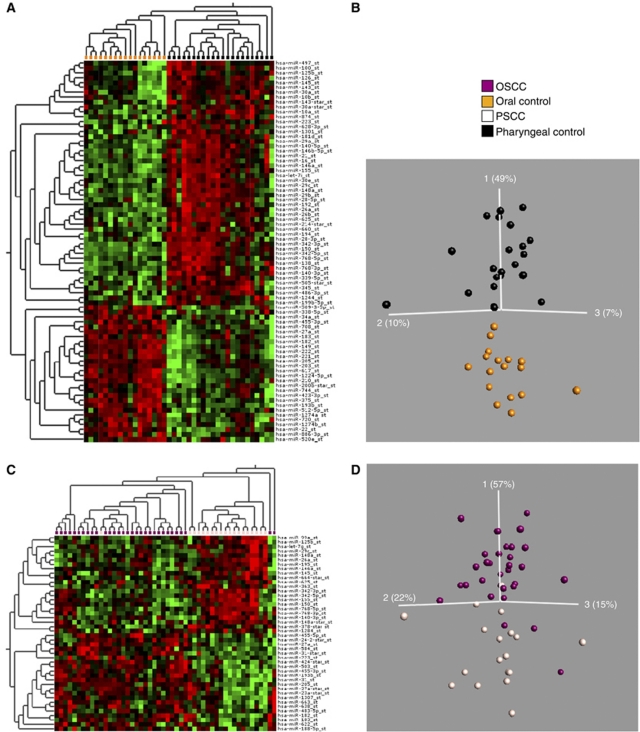
Visualisation of differentially expressed miRs between oral cavity samples and pharyngeal samples. (**A** and **B**) Control samples. (**A**) Heatmap: Oral cavity samples (gold) and pharyngeal samples (black). (**B**) PCA plot: Oral cavity samples (gold) and pharyngeal samples (black). (Subset of variables *P*<0.001, *q*<0.01.) (**C** and **D**) SCC samples: (**C**) Heatmap: PSCC samples (off-white) and OSCC samples (purple). (**D**) PCA plot: PSCC samples (off-white) and OSCC samples (purple) (*P*<0.01, *q*<0.13).

As shown in the PCA plot, OSCC and PSCC samples were more similar to each other compared with the normal epithelia (Figure 1). In a direct comparison of oral and pharyngeal tumours (Figure 5), 46 miRNAs were found to be differentially expressed (*P*<0.01 and an FDR *q*<0.13). Twenty-one miRNAs were upregulated and 25 miRNAs were downregulated. None of the miRNAs were exclusive to the groups and the fold changes were moderate. Two samples did not cluster correctly according to their anatomical subsite, of which one was in the soft palate just on the border of oral cavity and pharynx and one sample was an oral HPV-positive SCC. Comparing PSCC with normal pharyngeal epithelium revealed only 38 differentially expressed miRNAs (*P*<0.01, *q*<0.2) (Supplementary Figure 1). Twenty of these were also perturbed in the OSCC (Supplementary Figure 2). Within the PSCC samples, we moreover compared the HPV-positive and -negative cancers. However, when the samples were selected based on P16^INK4A^ immune or ISH staining alone, there was no difference between the groups, but a clear separation was seen with HPV PCR. However, if two of the three criteria were positive, as described above, the optimal separation between the HPV-positive and -negative groups was found (see Figure 4), and a number of miRNAs turned out to be differentially expressed (Table 4). The most upregulated miRNA was miR-363 and the most downregulated miRNAs were miR127-3p and miR-379, which exhibited fold changes of 1.6, −2.5 and −2, respectively (*P*<0.02, *q*<0.5).

Although OSCC and PSCC were partially similar (Figure 1), we observed that fewer miRNAs (114 *vs* 38) differed among pharyngeal cancers and the normal pharyngeal epithelium, indicating that miRNAs were different in the two epithelial linings. Consequently, we compared the miRNA composition in the two anatomical locations (Figure 5). The signature differed and 127 miRNAs distinguished the two epithelia (*P*<0.01, *q*<0.06). Fifty-six were upregulated and 71 were downregulated, respectively, in the normal oral *vs* pharyngeal epithelium. There was almost a 10-fold less miR-150 and a fivefold more miR-1224 and miR-375 expression in the oral epithelium. This suggests that miRNAs may be important for maintaining distinct phenotypes of oral epithelium in the oral cavity and pharynx. The trend towards different expression profiles in patients with and without lymph node metastases was not observed in PSCC samples (as opposed to the OSCC samples). In addition, no differences in the miRNA expression were observed when comparing histological differentiation, stage or smoking status.

Finally, five miRNAs and 20 samples were selected for validation by qPCR. MicroRNA-125b and miR-181b were used only for oral cavity samples comparing oral cavity cancer samples (*n*=5) with oral cavity control samples (*n*=5). MicroRNA-375, miR-187 and miR-31 were used for qPCR in comparing all four groups (OSCC (*n*=5), oral cavity controls (*n*=5), PSCC (*n*=5) and pharyngeal controls (*n*=5)). The results of the qPCR validation are shown in Supplementary Figure 3. In all cases, qPCR results confirmed the array data.

### Generation of a diagnostic classifier for oral cancer

As the analysis of differentially expressed miRNAs provided a putative signature for oral carcinogenesis, we generated a classifier that could differentiate between normal epithelium and malignant lesions. The classifier was based on the SVM algorithm developed in our R script. We compared the accuracy of the prediction by using the different classification methods implemented in BRB-Array tools. We selected 61 miRNAs to discriminate between the normal oral biopsies and OSCCs. Basically all the employed algorithms provided accurate predictions. The cross-validation misclassification rate using the LOOCV procedure was 0% for all the tested *P*-value cutoff. The composition of the classifier is shown in Supplementary Table 1. The diagnostic signature was moreover tested on an independent validation material consisting of seven OSCC biopsies and seven biopsies with normal epithelium. Collection of biopsies for the test set as well as the RNA isolation, hybridisation to array and all analyses were performed completely independent of the training set. In the test set, one normal sample was falsely classified as cancer, resulting in an accuracy of 93% (13 out of 14) and a sensitivity and specificity, respectively, of 100% (7 out of 7) and 86% (6 out of 7). The predicted probability of the new samples belonging to the class of cancer/normal epithelium is shown in Supplementary Table 2.

We conclude that miRNAs signatures are efficient in the diagnosis of oral cancer.

### Gene expression in oral cancer

One thousand eight hundred eighty-three genes were differentially expressed between oral cancer and oral controls with an adjusted *P*-value (FDR *q*<0.05, *P*<0.007, variance 0.07). The most significant aberrations were an 87-fold upregulation of POSTN (periostin, osteoblast-specific factor), 83-fold upregulation of MMP1 (matrix metallopeptidase 1 (interstitial collagenase)) and a 56-fold downregulation of TMPRSS11B (transmembrane protease, serine 11B), as well as a 27-fold downregulation of KRT4 (keratin4). The 20 most up- and downregulated genes are listed in Supplementary Table 3 and a heatmap of differentially expressed genes between the two groups are shown in Supplementary Figure 4. The significant differentially expressed genes (*P*<0.007, *q*<0.05, fold change >2/−2) were imported into the Ingenuity Systems Pathway Analysis and a gene set enrichment analysis (Core IPA) was made to define pathways or biological functions, which have a likelihood of being regulated in the oral cavity cancers. Summary of core pathway analysis revealed that the following molecular and cellular functions were most significantly enriched in the gene list: cellular movement, cell morphology, cellular growth and proliferation, cell-to-cell signalling and interaction together with cellular development. One of the top canonical pathways was, as in the miRNA predicted pathways, the TGF*β* signalling. On the basis of the biological functions that were enriched in the regulated gene list, the software proposes the diseases where these biological functions may be affected. The proposed diseases were as follows: connective tissue disorder, genetic disorder and cancer. Supplementary Tables 5–10 illustrate the summary of the analysis.

### miRNA target prediction and gene ontology analysis

From the miRNA that changed more than fivefold up or down between OSCC and oral controls, we found 452 predicted target genes, which had a combined weighted inhibitor score of more than 2/−2. Fifty genes with the highest and lowest combined score as well as their matching fold changes identified in the gene expressions analysis are shown in Supplementary Table 4. As shown in the table, no systematic correlation between the weighted combined score and the matching fold changes in the gene expressions from the same samples could be found. The 452 genes with a combined score of 2/−2 were used for the core analysis of the functions of the predicted target genes in Ingenuity Systems Pathway Analysis. The analysis of predicted target genes displayed distinct molecular and cellular functions. In summary, cell cycle, cell death, cellular growth and proliferation, as well as post-translational modification were the most prominent features. The top canonical pathways were mTOR signalling, HGF–, TGF*β* and IGF-1 signalling. From these biological functions, a suggestion of disease was made and satisfactorily the number one predicted disease was cancer. Supplementary Tables 11–16 illustrate the summary of the analysis.

## Discussion

Even though advances in diagnostic tools and treatments have been achieved over the past three decades, the mortality of head and neck cancer patients is still high and the offered treatment modalities are still associated with major adverse effects with considerable impact in the quality of life (Corry *et al*, 2010). HNSCC comprises a group of diverse heterogeneous cancers that develop from many different anatomical sites and are associated with different risk factors related to both behaviour and genetic background (Marcu and Yeoh, 2009). Clinical features and prognosis are likewise very different, and it would be of great importance to obtain site-specific miRNA signatures to verify origin of the tumour and prognostic values. For example, HPV status greatly influences the clinical features and prognosis (Lajer and Buchwald, 2010). This emphasises the importance of using larger patient material for profiling analysis as cell lines may not fully reflect the miRNA profiles of solid tumours and may be inadequate to illustrate the diversity among the different HNSCC.

Barker *et al* (2009) used miRNA expression profiles to identify unknown primary lesions in the head and neck region for three distinct subsites, that is, the tonsils, base of tongue and post-nasal space (Barker *et al*, 2009). However, in another study on formalin-fixed paraffin-embedded specimens, Hui *et al* (2010) did not see any difference in the expression profiles of three subsites, that is, the hypopharynx, the oropharynx and the larynx. This underlines the importance of further analysis, legalising this study.

There is a considerable variation in the miRNA profiles across the various miRNA profile studies of head and neck cancer. Differences may reflect both technically issues and differences in stage, grade and sampling from multiple anatomical sites as well as the use of cell lines.

We performed a prospective translational and multidisciplinary study comprising a large and clinically well-defined patient material, which allowed us to generate site-specific miRNA expression profiles from oral cavity and pharyngeal cancers, as well as determine the influence of HPV on miRNA expression profile in head and neck cancers.

The oral cavity samples showed very consistent expression profiles and benign and malignant samples separated clearly even in the uncensored PCA plots. Homogeneity was particularly striking among the OSCC and oral cavity control samples, whereas the PSCC and pharyngeal control samples were more diverse. One of the reasons for this may be a higher contamination of non-tumour cells in the biopsies from the PSCC patients, which are much more difficult to examine and perform biopsies from, compared with oral cavity cancers, which normally present themselves as a clear visible tumour easy to distinguish from surrounding structures. The difference between cancer samples and controls was also less pronounced in the pharynx. Many of the miRNAs lost in oral cancers and highly expressed in oral controls were more highly expressed in the pharyngeal cancers and only slightly higher expressed in the pharyngeal controls, indicating a higher resemblance in miRNA profile of pharyngeal epithelium with cancer. We also noted a marked difference in the miRNA expression between the controls from the oral cavity and pharynx. This may be related to the higher cell turnover of pharyngeal mucosa than oral mucosa (Squier *et al*, 1976). In addition, the mucosa from the oral cavity has an ectodermic origin, whereas the pharyngeal mucosa is of endodermic origin. The considerable difference between the oral cavity and pharynx in the expression profiles of both malignant and control tissue emphasises the importance of examining the different anatomical sites separately.

In this study, we found a considerable number of differentially regulated miRNA in the cancer tissue compared with the controls. Although a number of the former has been identified in other profile studies, a large fraction has not been described in head and neck cancer before. In the comprehensive profile study of 51 tumour samples of the oral cavity, pharynx and larynx by Hui *et al* (2010), only 10 miRNAs (miR-93, miR-25, miR-155, miR-21, miR-let7i, miR-223, miR-99a, miR100, miR-125b, miR-375) from a total of 322 examined miRNAs were identical to the ones found in this study.

The most differentially regulated miRNAs in this study was miR-375, which was highly expressed in oral controls and almost absent in the OSCCs, but the difference between cancer and control was much less pronounced in the pharynx. This finding is in accordance with a previous report (Avissar *et al*, 2009) showing that miR-375 expression was higher in tumours of pharyngeal and laryngeal origin compared with oral tumours. MicroRNA-375 is involved in the glucose regulation of insulin gene expression and cell growth, decreasing the cell's sensitivity to insulin. Malignant cells, and especially those originating from head and neck cancers, have a high glucose uptake, so an upregulation of glucose transporters is in accordance with a high sensitivity to insulin that is not blocked by miR-375. In accordance with this, four glucose transporters were upregulated in our gene arrays (SLC2A1, SLC2A3, SLC2A9 and SLC2A14).

MicroRNA-31 was significantly upregulated in the OSCC samples. This has also been shown in OSCC tissue, saliva and blood in other studies (Liu *et al*, 2010), as well as in cervical cancer, lung cancer and colorectal cancers (Muralidhar *et al*, 2007).

MicroRNA-187 was upregulated in both OSCC and PSCC, and has also been found to be upregulated in other malignant tumours, for example, thyroid cancer (Nikiforova *et al*, 2008). Previously, identification of miR-181b, miR-21 and miR-345 in oral leukoplakia has indicated that these miRNAs may be part of progression to malignancy (Cervigne *et al*, 2009). All these miRNA were upregulated in the OSCC samples. MicroRNA-181b was also found to be more highly expressed in patients with lymph node metastases from oral cavity cancer. These observations strongly support that these miRNAs may have diagnostic as well as prognostic value. In addition, we found a significant downregulation of miR-125b and miR-100. MicroRNA-125b is frequently downregulated in a number of malignancies, including breast and prostate cancers (Shi *et al*, 2008; O’Day and Lal, 2010). In breast cancer, miR-125b is downregulated and it may function as a tumour suppressor. When over expressed in breast cancer cell lines, miR-125b suppresses HER2 and HER3 mRNA and protein levels leading to a reduction in anchorage-dependent growth, cell motility and invasiveness (O’Day and Lal, 2010). Furthermore, this miRNA is reported to target p53 and several of the upstream regulators (Zhang *et al*, 2009).

Several genetic aberrations are characteristic of OSCC, with amplification and deletions of chromosomal band 11q13 and loss of distal 11q being among the most prevalent. MicroRNA-100 and miR-125 are closely located at the distal part of chromosome 11q. Loss of heterozygosity has been reported in over 60% of HNSCC at 11q and several oncogenes and tumour suppressor genes are located at chromosome 11 (Scully *et al*, 2000). It is hypothesised that the expression of miRNAs mapping to 11q is altered in OSCC because of loss or amplification of chromosomal material, and that this contributes to the development and progression of OSCC. In OSCC tumours and cell lines, it was found that miR-125b and miR-100 expression were downregulated and that transfecting cells with exogenous miR-125b and miR-100 significantly reduced cell proliferation and modified the expression of target and non-target genes, including some that are overexpressed in radio-resistant OSCC cells (Henson *et al*, 2009). Interestingly, although the one control sample was misclassified as a cancer sample in the test set of the classifier, it showed high levels of both miR-125b and miR-100.

Despite the fact that no direct correlation could be seen between the miRNA expression and the gene expression, the analysis of predicted target genes of the most significantly changed miRNAs pointed at a series of target mRNA encoding proteins involved in cancer. There was no consistent and significant change in the mRNA expression of genes that contained onco-miR or tumour suppressor miR target sites, indicating that target protein translation rather than mRNA cleavage may be the prominent feature. In addition, other studies, recording global analysis of the relationship between miRNA and the proteome, found that single miRNA expression alterations only had modest impact on individual protein expression (Baek *et al*, 2008; Selbach *et al*, 2008). Furthermore, Gallagher *et al* (2010) suggested that the collective changes in many miRNAs may be the most biologically interesting parameter to consider and they hypothesised that it is most likely that miRNAs work cooperatively *in vivo* and physiological regulation of a single protein by a single miRNA may be a rather rare occurrence. In addition, evidence of cleavage of mRNA as a result of miRNA binding originates from cell line studies, where a large change in one miRNA is created by transfecting or by knock down. This may be far from the conditions *in vivo*. In the study from Gallagher *et al* (2010), they did not find any correlation between the miRNA expression and the genome expression either.

Even though there was no correlation with miRNA profile and stage or grade, a distinct miRNA expression pattern was associated with lymph node metastasis in the oral cavity cancer group. MicroRNA-181b was upregulated and miR-942 and miR-181c^*^ were downregulated, respectively. The number of patients with lymph node metastasis in this study is too low to make a clear signature and the changes were moderate as described. Additional investigation in a prognostic study is obviously needed. Biomarkers that predict primary tumours with metastatic potential are of clinical importance, as spread of tumour cells to the regional lymph nodes in the neck is the most important independent prognostic factor in head and neck cancer patients. The presence of lymph node metastases in HNSCC patients at the time of diagnosis reduces the survival to 50% compared with patients without nodal involvement (N0). In addition, nodal metastasis occurs in 20–50% of patients with clinically N0 head and neck cancer. Treating the neck with either surgical neck dissection or radiotherapy is accompanied with significant side effects, and the treatment strategy of cervical lymph nodes is still under debate (von Buchwald *et al*, 2002).

Human papilloma virus-positive HNSCC appears to be different from HPV-negative HNSCC in both molecular and clinical features (Lajer and Buchwald, 2010). In general, it is recommended that at least two standardised and recognised methods should be used to confirm the diagnosis of clinically relevant HPV-positive HNSCC. This is underscored in this project as a clear separation in the PCA plots and heatmaps could only be achieved when at least two out of three positive criteria were met. Human papilloma virus status clearly has an influence on the miRNA expression profiles.

A number of miRNAs were perturbed in the HPV-positive pharyngeal SCC samples compared with the HPV-negative pharyngeal SCC. The observed fold changes were as described modest and the FDR relatively high. This is at least partly due to a prominent infiltration of normal tissue in the HPV-negative PSCC and low number of HPV-positive samples. MicroRNA-145, miR-125a and miR-126, which are reduced in the HPV-positive PSCC, are also downregulated in cervical cancer tissue (Wang *et al*, 2008) and miR-126 has been suggested to function as a tumour suppressor in gastric and colorectal cancers (Feng *et al*, 2010; Li *et al*, 2010). MicroRNA-145 has been shown to be crucial for the gut development and epithelial maturation (Zeng *et al*, 2009). Notably, a recent study showed that miR-125a-5p can inhibit migration and invasion of lung cancer cells (Wang *et al*, 2009) and transfection of miR-125a-5p in an intestinal epithelial cell line decreased growth rate and transepithelial resistance of the cells (Dalmasso *et al*, 2010).

MiR-363 was elevated in the HPV-positive PSCC samples. This is in concordance with a most recent study, in which HPV-positive head and neck cancer cell lines showed a considerable upregulation of miR-363 and an siRNA knockdown of HPV16 E6 was accompanied by a reduction of miR-363 levels (Wald *et al*, 2010). MicroRNA-127-3p and miR-379 were both downregulated among the HPV-positive PSCC. These two miRNAs have been found to be differentially expressed in smokers *vs* non-smokers in patients with malignant mesothelioma and this may reflect the limited number of smokers in the HPV-positive PSCC group. The present material comprised only had five tonsillar SCC. To make a clear HPV miRNA classifier, it probably needs to include micro-dissected formalin-fixed paraffin-embedded tonsillar specimens to ensure a high proportion of HPV-positive samples and even tissue specificity.

We finally examined whether or not the distinct miRNA signatures could provide a solid basis for diagnostic classification between normal epithelium and malignant lesions in the oral cavity. A robust classifier was generated for oral cavity cancer, with an accuracy of 93% in the test samples. The test sample that failed was obtained from a patient who had clear inflammation in the area where the biopsy was taken. This sample clearly clustered with cancer samples and it may indicate that there are some similarities in the miRNA profiles between cancer and inflammation. Even though the test set was small, the classifier seemed robust, but needs to be confirmed in a larger validation study. A validation study should preferably also include inflamed tissue, which seems to have a degree of resemblance with malignant tissue as indicated above.

In conclusion, we found significant differences in miRNA expression profiles of malignant and benign tissue samples as well as remarkable site-specific differences. In addition, the presence of lymph node metastasis at the time of diagnosis in oral SCC seems to influence the miRNA expression. Human papilloma virus infection in pharyngeal SCC shows a distinct miRNA expression profile, but needs to be further elucidated in a specific tonsillar SCC material.

## Figures and Tables

**Table 1:** Patient characteristics

	**OSCC patients**	**Oral cavity control patients**	**PSCC patients**	**Pharyngeal control patients**	**Total**
Number of patients	30	17	19	22	88
					
*Gender*					
Female	15	10	6	16	47
Male	15	7	14	6	42
					
*Age*					
Median	61	62	61	36	60
					
*Smoking status*					
Never smoked	8	6	8	10	32
Former smoker	2	1	2	2	7
Current smoker	18	8	9	10	45
ND	2	2	0		4

Abbreviations: ND=not determined; OSCC=oral squamous cell carcinoma; PSCC=pharyngeal squamous cell carcinoma.

**Table 2:** Tumour and normal tissue biopsy characteristics

	**OSCC**	**Oral cavity controls**	**PSCC**	**Pharyngeal controls**	**Total**
*Primary tumour location*					
Tongue	15	8			23
Bucca	2	8			10
Floor of mouth	7	1			8
Gingiva	5				5
Other oral cavity	1				1
Tonsil			5	14	19
Base of tongue			10	1	9
Other pharynx			4	7	11
					
*Stage*					
I	12		1		13
II	8		4		12
III	4		7		11
IVa and IVb	6		5		11
					
*Grade*					
Well differentiated	5		2		7
Intermediate differentiated	12		7		19
Poorly differentiated	13		10		23
p16 positive	3	0	10	0	13
*In situ* hybridisation positive	1	0	6	0	7
PCR HPV positive	3	0	6	1	9
HPV positive by two parameters	1	0	8	0	9

Abbreviations: HPV=human papilloma virus; ND=not determined; OSCC=oral squamous cell carcinoma; PCR=polymerase chain reaction; PSCC=pharyngeal squamous cell carcinoma.

**Table 3:** OSCC compared with oral cavity controls

**Probeset name**	***P*-value**	***q*-value (FDR)**	**Fold change SCC/control**
hsa-miR-375_st	1.35E-16	3.49E-14	−20
hsa-miR-1224-5p_st	4.48E-18	3.47E-15	−11.1
hsa-miR-617_st	8.22E-17	3.19E-14	−8.3
hsa-miR-99a_st	1.13E-12	2.18E-10	−5.3
hsa-miR-125b_st	4.8E-07	0.0000233	−2.3
hsa-miR-378_st	2.09E-06	0.0000676	−2.1
hsa-miR-27b_st	1.14E-06	0.0000422	−2
hsa-miR-125b-2-star_st	4.14E-06	0.000107	−2
hsa-miR-21-star_st	3.14E-07	0.0000162	2
hsa-miR-345_st	6.68E-06	0.000157	2.1
hsa-miR-106b-star_st	2.58E-06	0.0000768	2.2
hsa-miR-132_st	6.91E-07	0.0000297	2.3
hsa-miR-27a-star_st	5.35E-10	5.93E-08	2.4
hsa-miR-181b_st	8.05E-08	0.00000567	2.4
hsa-miR-181a_st	1.54E-07	0.00000918	2.4
hsa-miR-424-star_st	7.77E-10	7.53E-08	2.9
hsa-miR-155_st	8.41E-07	0.0000326	2.9
hsa-miR-146a_st	7.57E-07	0.0000309	3
hsa-miR-146b-5p_st	1.26E-07	0.00000816	3.1
hsa-miR-1246_st	4.53E-08	0.00000351	3.6
hsa-miR-187_st	2.02E-09	0.000000174	3.8
hsa-miR-503_st	1.81E-12	2.81E-10	4.2
hsa-miR-223_st	2.25E-06	0.0000698	4.26
hsa-miR-21_st	9.2E-11	1.19E-08	5.6
hsa-miR-31_st	1.79E-06	0.000063	6.85

Abbreviations: FDR=false discovery rate; miRNA=microRNA; OSCC=oral squamous cell carcinoma.

Top 25 significant differentially expressed miRNAs with fold change of more than 2.

**Table 4:** List of 21 differentially expressed miRNAs with a fold change of more than 1.5 in HPV-positive PSCC compared with HPV-negative PSCC (*P*<0.02, *q*<0.5)

**Probeset name**	***P*-value**	***q*-value**	**Fold change**
hsa-miR-127-3p_st	0.01089	0.49	−2.5
hsa-miR-379_st	0.00077	0.228	−2.4
hsa-miR-125a-5p_st	0.00976	0.49	−2.1
hsa-miR-432_st	0.01914	0.49	−1.9
hsa-miR-143_st	0.00262	0.303	−1.9
hsa-miR-145_st	0.00144	0.228	−1.9
hsa-miR-409-5p_st	0.01931	0.49	−1.9
hsa-miR-433_st	0.01831	0.49	−1.9
hsa-miR-381_st	0.00128	0.228	−1.9
hsa-miR-199a-3p_st	0.01379	0.49	−1.8
hsa-miR-199b-3p_st	0.01523	0.49	−1.8
hsa-miR-26b_st	0.0092	0.49	−1.8
hsa-miR-199b-5p_st	0.01441	0.49	−1.7
hsa-miR-143-star_st	0.00168	0.228	−1.7
hsa-miR-1201_st	0.00927	0.49	−1.7
hsa-miR-126_st	0.00038	0.228	−1.7
hsa-miR-409-3p_st	0.0156	0.49	−1.6
hsa-miR-101_st	0.00698	0.49	−1.6
hsa-miR-517a_st	0.01038	0.49	−1.5
hsa-miR-195-star_st	0.01467	0.49	1.5
hsa-miR-363_st	0.01581	0.49	1.6

Abbreviations: HPV=human papilloma virus; miRNA=microRNA; PSCC=pharyngeal squamous cell carcinoma.
