# Supplementary material for: Different miRNA signatures of oral and pharyngeal squamous cell carcinomas: a prospective translational study
Source: Br J Cancer. 2011 Feb 15;104(5):830–40. doi: 10.1038/bjc.2011.29 (PMC3048216; doi:10.1038/bjc.2011.29)
Supplement: Supplementary Information [file bjc201129x1.doc]

Supplementary


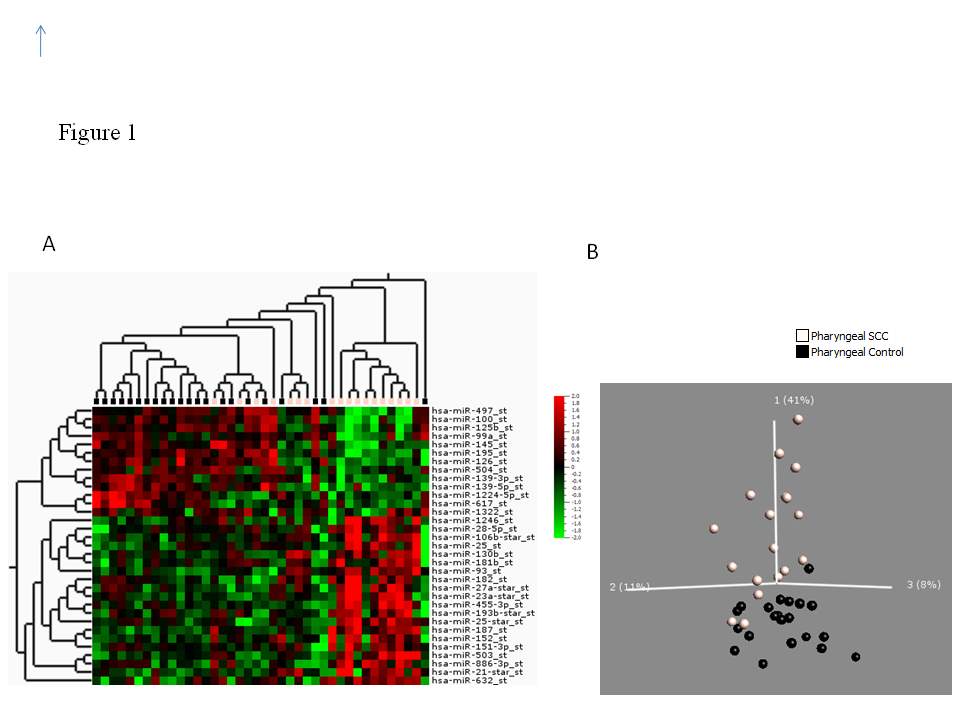


Figure 1: Visualisation of differentially expressed miRs between PSCC and pharyngeal controls. A) Heat map: PSCC (off-white) and controls (black). B) PCA plot: PSCC (off-white) versus pharyngeal controls (black). P<0.01, q<0.1


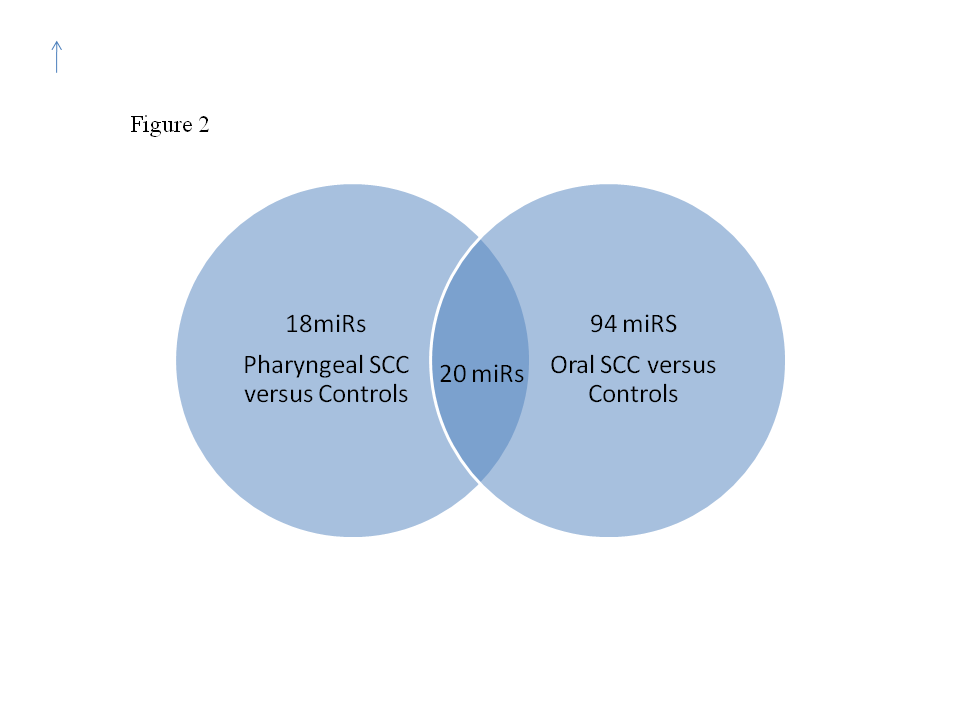
 Figure 2: miRNAs shared by both cancer groups (OSCC & PSCC)


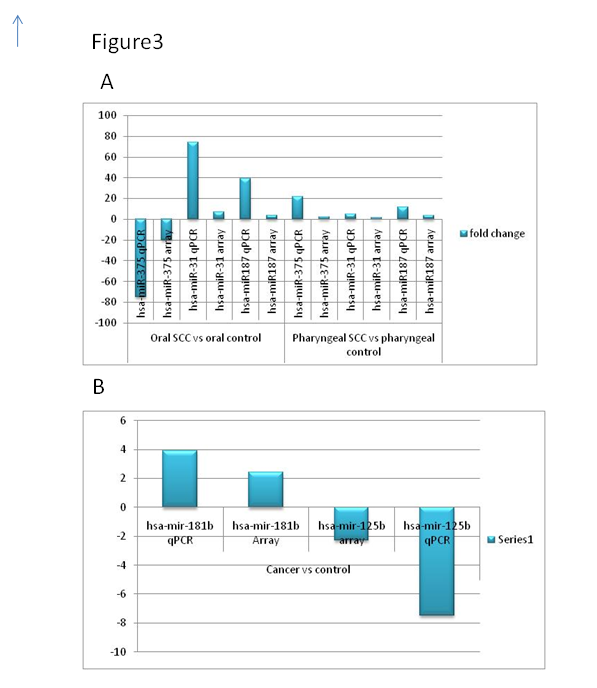
 Figure 3: A) qPCR (and full array data) of hsa-miR-375, hsa-miR-187 and hsa-miR-31 in 20 patients. i.e 5 from each group illustrating fold changes. B) Fold changes qPCR (and full array data) of hsa-miR-181b and has-miR-125b in oral cavity cancer (n=5) compared to oral cavity control patients (n=5). The level of mature miRNA was determined relative to that of hsa-miR-191.

| **Parametric p-value** | **t-value** | **% CV support** | **Geom mean of intensities in class 1** | **Geom mean of intensities in class 2** | **Fold-change** | **Unique id** |
| --- | --- | --- | --- | --- | --- | --- |
| < 1e-07 | -13,708 | 100 | 27.77 | 294.86 | 0.094 | hsa-miR-1224-5p_st |
| < 1e-07 | -13,357 | 100 | 13.33 | 104.39 | 0.13 | hsa-miR-617_st |
| < 1e-07 | -13.28 | 100 | 17.87 | 323.38 | 0.055 | hsa-miR-375_st |
| < 1e-07 | -9,203 | 100 | 113.16 | 566.04 | 0.2 | hsa-miR-99a_st |
| 2.1e-06 | -5,393 | 100 | 3171.45 | 4681.74 | 0.68 | hsa-miR-23b_st |
| 2.4e-06 | -5,348 | 100 | 591.6 | 1259.71 | 0.47 | hsa-miR-125b_st |
| 3.9e-06 | -5,212 | 100 | 386.95 | 767.26 | 0.5 | hsa-miR-27b_st |
| 4.5e-06 | -5,173 | 100 | 25.03 | 45.19 | 0.55 | hsa-miR-125b-2-star_st |
| 9.7e-06 | -4,945 | 100 | 24.44 | 32.26 | 0.76 | hsa-miR-129-3p_st |
| 1.74e-05 | -4,773 | 100 | 257.35 | 526.17 | 0.49 | hsa-miR-378_st |
| 2.78e-05 | -4,633 | 100 | 1010.28 | 3618.78 | 0.28 | hsa-miR-203_st |
| 4.29e-05 | -4,502 | 100 | 16.19 | 22.11 | 0.73 | hsa-miR-512-5p_st |
| 5.76e-05 | -4,413 | 100 | 10.67 | 14.33 | 0.74 | hsa-miR-1257_st |
| 9.38e-05 | -4,263 | 100 | 62.02 | 138.96 | 0.45 | hsa-miR-720_st |
| 0.0001219 | -4,182 | 100 | 26.12 | 32.65 | 0.8 | hsa-miR-187-star_st |
| 0.0002944 | -3,905 | 100 | 128.87 | 240.94 | 0.53 | hsa-miR-34a_st |
| 0.0004422 | -3,774 | 100 | 43.83 | 58.16 | 0.75 | hsa-miR-29b-2-star_st |
| 0.0006651 | 3,641 | 100 | 950.46 | 681.48 | 1.39 | hsa-miR-93_st |
| 0.0006573 | 3,644 | 100 | 33.7 | 22.88 | 1.47 | hsa-miR-625_st |
| 0.0005221 | 3.72 | 100 | 30.83 | 21.23 | 1.45 | hsa-miR-128_st |
| 0.0003356 | 3,863 | 100 | 5340.18 | 4507.53 | 1.18 | hsa-miR-24_st |
| 0.0002445 | 3,964 | 100 | 35.76 | 22.93 | 1.56 | hsa-miR-330-3p_st |
| 0.0002132 | 4,007 | 100 | 121.89 | 67.75 | 1.8 | hsa-miR-130b_st |
| 0.0001918 | 4.04 | 100 | 21.82 | 13.76 | 1.59 | hsa-miR-214-star_st |
| 0.0001076 | 4,221 | 100 | 27.36 | 17.99 | 1.52 | hsa-miR-584_st |
| 9.81e-05 | 4,249 | 100 | 496.95 | 254.46 | 1.95 | hsa-miR-455-3p_st |
| 9.51e-05 | 4,259 | 100 | 500.72 | 378.24 | 1.32 | hsa-miR-151-5p_st |
| 8.41e-05 | 4,297 | 100 | 22.71 | 12.24 | 1.86 | hsa-miR-1290_st |
| 5.37e-05 | 4,434 | 100 | 35.59 | 21.15 | 1.68 | hsa-miR-31-star_st |
| 4.31e-05 | 4,501 | 100 | 224.27 | 119.55 | 1.88 | hsa-miR-25_st |
| 2.39e-05 | 4,678 | 100 | 72.52 | 39.41 | 1.84 | hsa-miR-1307_st |
| 1.93e-05 | 4,743 | 100 | 30.89 | 15.3 | 2.02 | hsa-miR-25-star_st |
| 1.85e-05 | 4,755 | 100 | 56.59 | 27.93 | 2.03 | hsa-miR-23a-star_st |
| 1.17e-05 | 4,891 | 100 | 36.79 | 24.43 | 1.51 | hsa-miR-331-3p_st |
| 1.14e-05 | 4,898 | 100 | 35.97 | 16.48 | 2.18 | hsa-miR-1301_st |
| 5.3e-06 | 5,121 | 100 | 65.12 | 31.8 | 2.05 | hsa-miR-345_st |
| 4.4e-06 | 5,175 | 100 | 58.6 | 31.62 | 1.85 | hsa-miR-339-5p_st |
| 3.7e-06 | 5,228 | 100 | 110.15 | 26.85 | 4.1 | hsa-miR-223_st |
| 3.3e-06 | 5,264 | 100 | 38.22 | 21.78 | 1.75 | hsa-miR-505-star_st |
| 2.9e-06 | 5,301 | 100 | 77.24 | 48.24 | 1.6 | hsa-miR-28-3p_st |
| 2.5e-06 | 5,343 | 100 | 781.52 | 430.36 | 1.82 | hsa-let-7i_st |
| 2.4e-06 | 5,358 | 100 | 46.67 | 25.75 | 1.81 | hsa-miR-342-5p_st |
| 2.2e-06 | 5,378 | 100 | 31.02 | 17.84 | 1.74 | hsa-miR-181d_st |
| 2.2e-06 | 5.38 | 100 | 83.7 | 38.54 | 2.17 | hsa-miR-106b-star_st |
| 1.00E-06 | 5,605 | 100 | 88.53 | 45.82 | 1.93 | hsa-miR-28-5p_st |
| 7.00E-07 | 5.69 | 100 | 2484.89 | 364.66 | 6.81 | hsa-miR-31_st |
| 6.00E-07 | 5,733 | 100 | 56.38 | 36.86 | 1.53 | hsa-miR-92b_st |
| 4.00E-07 | 5,896 | 100 | 81.01 | 35.52 | 2.28 | hsa-miR-132_st |
| 4.00E-07 | 5,901 | 100 | 507.46 | 222.09 | 2.28 | hsa-miR-181a_st |
| 3.00E-07 | 5,963 | 100 | 131.36 | 38.63 | 3.4 | hsa-miR-1246_st |
| 2.00E-07 | 6,032 | 100 | 201.48 | 60.35 | 3.34 | hsa-miR-146a_st |
| 2.00E-07 | 6,049 | 100 | 398.46 | 134.1 | 2.97 | hsa-miR-155_st |
| 1.00E-07 | 6,196 | 100 | 25.58 | 14.62 | 1.75 | hsa-miR-181c_st |
| < 1e-07 | 6,295 | 100 | 53.08 | 26.01 | 2.04 | hsa-miR-21-star_st |
| < 1e-07 | 6,355 | 100 | 95.35 | 28.94 | 3.29 | hsa-miR-146b-5p_st |
| < 1e-07 | 6,363 | 100 | 265.21 | 109.43 | 2.42 | hsa-miR-181b_st |
| < 1e-07 | 7,434 | 100 | 41.89 | 15.32 | 2.73 | hsa-miR-424-star_st |
| < 1e-07 | 7,659 | 100 | 46.33 | 12.81 | 3.62 | hsa-miR-187_st |
| < 1e-07 | 7,909 | 100 | 61.7 | 25.54 | 2.42 | hsa-miR-27a-star_st |
| < 1e-07 | 8,083 | 100 | 349.26 | 60.33 | 5.79 | hsa-miR-21_st |
| < 1e-07 | 9,246 | 100 | 59.05 | 14.79 | 3.99 | hsa-miR-503_st |

Table 1: Composition of classifier:

| **Array id** | **Probability** |
| --- | --- |
| **85vs** | 1 |
| **86vs** | 1 |
| **91vs** | 1 |
| **109vs** | 1 |
| **110vs** | 1 |
| **117vs** | 1 |
| **131vs** | 1 |
| **141vs** | p < 1.0e-3 |
| **142vs** | p < 1.0e-3 |
| **143vs** | 0.997 |
| **144vs** | p < 1.0e-3 |
| **145vs** | p < 1.0e-3 |
| **146vs** | p < 1.0e-3 |
| **147vs** | 0.003 |

Table 2: Predicted probability of each new sample belonging to the class (cancer) from the Bayesian Compound Covariate. The misclassified sample is colored yellow.

| **Gene Symbol** | **p-Value** | **q-Value** | **Fold change** |
| --- | --- | --- | --- |
| POSTN | 7.02E-09 | 0.0001806 | 86.7 |
| COL12A1 | 8.52E-06 | 0.0059248 | 18.1 |
| LAMC2 | 9.68E-07 | 0.0019724 | 15.9 |
| TNC | 2.88E-06 | 0.0039906 | 15.1 |
| MMP13 | 9.42E-05 | 0.0140257 | 14.8 |
| PTGS2 | 3.14E-05 | 0.0097952 | 11.1 |
| ADAM12 | 9.97E-07 | 0.0019724 | 9.1 |
| PXDN | 2.91E-05 | 0.0094765 | 8.6 |
| COL1A1 | 5.36E-08 | 0.0004599 | 8.3 |
| COL6A3 | 5.20E-07 | 0.0019724 | 7.9 |
| COL1A2 | 7.87E-07 | 0.0019724 | 7.5 |
| FST | 3.88E-06 | 0.0039906 | 7.3 |
| ITGA5 | 3.43E-05 | 0.0098545 | 6.7 |
| VCAN | 1.90E-05 | 0.0078783 | 6.7 |
| IGF2BP2 | 8.75E-05 | 0.0137743 | 6.6 |
| COL3A1 | 3.05E-05 | 0.0097908 | 6.3 |
| SPARC | 4.60E-07 | 0.0019724 | 5.7 |
| TGFBI | 6.98E-05 | 0.0126496 | 5.1 |
| LOXL2 | 1.60E-05 | 0.0074921 | 4.9 |
| COL5A2 | 1.81E-06 | 0.0030508 | 4.7 |
| PITX2 | 1.92E-05 | 0.0078783 | -2.7 |
| DHRS1 | 3.20E-05 | 0.0097952 | -2.8 |
| TTC22 | 5.14E-05 | 0.0111169 | -2.8 |
| NEBL | 3.28E-05 | 0.0097952 | -2.9 |
| PRSS22 | 0.0001026 | 0.0146259 | -3 |
| CAPN5 | 3.27E-05 | 0.0097952 | -3 |
| SHROOM3 | 5.30E-05 | 0.0113685 | -3.4 |
| FUT6 | 2.81E-05 | 0.0094765 | -3.4 |
| GALNT12 | 2.79E-05 | 0.0094765 | -3.5 |
| C2orf54 | 1.35E-05 | 0.0070903 | -3.7 |
| PRSS27 | 4.60E-05 | 0.0107201 | -3.7 |
| FAM3D | 8.17E-05 | 0.0133901 | -3.8 |
| FUT3 | 2.11E-05 | 0.0081694 | -4 |
| C1orf116 | 2.17E-05 | 0.0081694 | -4.1 |
| PPL | 3.84E-05 | 0.0098704 | -4.5 |
| SCNN1A | 0.000113 | 0.0149528 | -4.9 |
| PAX9 | 3.28E-05 | 0.0097952 | -5 |
| VSIG8 | 3.53E-05 | 0.0098545 | -5.2 |
| DUOXA2 | 3.33E-06 | 0.0039906 | -6.7 |
| DUOX2 | 1.09E-05 | 0.0065282 | -6.8 |

Table 3: Top 20 upregulated and downregulated genes in oral cavity SCC compared to Oral cavity control samples.


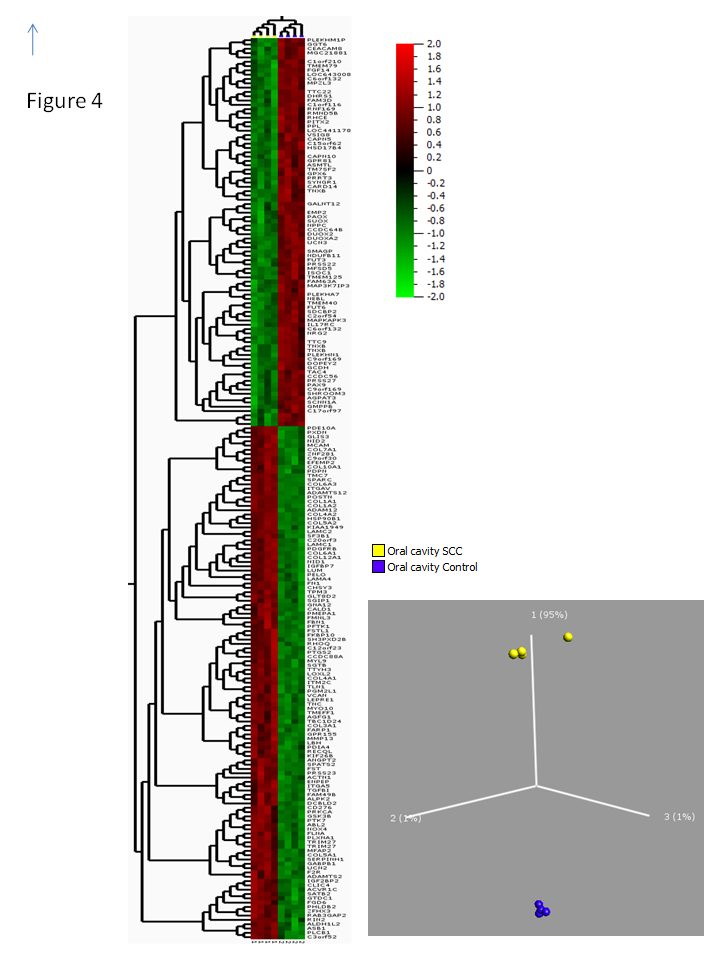


Figure 4: Visualisation of differentially expressed genes between OSCCand oral cavity controls. A) Heat map: OSCC (yellow) and controls (blue). B) PCA plot OSCC (yellow) versus oral cavityl controls (blue). Subset of variables, p<0.00015, q<0.015.

| **Gene Symbol** | **Down Reg. miRNAs** | **Up Reg.**  **miRNAs** | **Combined inhibitory score** | **p-value**  **(OSCC vs. Normal)** | **Fold-Change**  **(OSCC vs. Normal)** |
| --- | --- | --- | --- | --- | --- |
| PIK3C2A | 0 | -7.2 | -7.2 | 0.993447 | 1.00115 |
| KANK1 | 0 | -5.7 | -5.7 | 0.12478 | -1.62544 |
| JAZF1 | 0 | -5.7 | -5.7 | 0.0704236 | 1.26595 |
| NUMB | 0 | -5.5 | -5.5 | 0.748659 | 1.0484 |
| SLC2A3 | 0 | -5.4 | -5.4 | 0.0160333 | 5.11015 |
| TRAF6 | 0 | -5.3 | -5.3 | 0.128211 | -1.21502 |
| ZFP36L1 | 0 | -5.1 | -5.1 | 0.0321784 | 1.37521 |
| TIMP3 | 0 | -5.1 | -5.1 | 0.151486 | 2.05683 |
| TIMP3 | 0 | -5.1 | -5.1 | 0.176803 | 1.634 |
| GATAD2B | 0 | -5.1 | -5.1 | 0.428678 | 1.08995 |
| YLPM1 | 0 | -5.0 | -5.0 | 0.280989 | -1.07464 |
| RASA1 | 0 | -4.9 | -4.9 | 0.0347868 | 1.30185 |
| HISPPD1 | 0 | -4.8 | -4.8 | 0.818146 | -1.01827 |
| PLAG1 | 1.1 | -6.0 | -4.8 | 0.0165232 | 2.70177 |
| BRWD1 | 0.9 | -5.8 | -4.8 | 0.996818 | 1.00043 |
| PPP1R3B | 0.0 | -4.8 | -4.8 | 0.16721 | 1.10566 |
| WNK1 | 0.4 | -5.1 | -4.8 | 0.67105 | -1.06678 |
| ATP2B1 | 0.4 | -5.1 | -4.7 | 0.00698499 | 2.35344 |
| YOD1 | 0.3 | -4.8 | -4.6 | 0.0683626 | -1.62127 |
| EIF2C4 | 0.0 | -4.6 | -4.6 | 0.536419 | 1.15694 |
| LEMD3 | 0 | -4.6 | -4.6 | 0.209204 | 1.24802 |
| FBXW7 | 1.8 | -6.2 | -4.4 | 0.147878 | -1.48909 |
| JARID2 | 0.0 | -4.4 | -4.4 | 0.081284 | 1.30421 |
| ANKFY1 | 0.0 | -4.4 | -4.4 | 0.00675358 | 1.13005 |
| MANBA | 4.1 | 0.0 | 4.1 | 0.0500782 | 1.67221 |
| TSC1 | 4.1 | 0.0 | 4.1 | 0.403606 | -1.08069 |
| LDHB | 4.1 | 0.0 | 4.1 | 0.00245632 | 2.21291 |
| IKZF4 | 4.2 | 0.0 | 4.2 | 0.844271 | 1.01762 |
| SPTBN1 | 4.2 | 0.0 | 4.2 | 0.00523196 | 1.27662 |
| MAL2 | 4.2 | 0.0 | 4.2 | 0.139902 | -1.20392 |
| DVL3 | 4.2 | 0.0 | 4.2 | 0.0913742 | 1.49095 |
| KIAA0831 | 4.3 | 0.0 | 4.3 | 0.510382 | -1.07162 |
| OGFOD1 | 4.6 | 0.0 | 4.6 | 0.260334 | -1.14127 |
| SEC23A | 4.7 | 0.0 | 4.7 | 0.000669424 | 1.766 |
| IPO9 | 5.0 | -0.2 | 4.8 | 0.0234652 | 1.47042 |
| DCUN1D4 | 4.8 | 0.0 | 4.8 | 0.68494 | 1.09059 |
| MAP2K4 | 4.9 | 0.0 | 4.9 | 0.500279 | -1.05692 |
| AHR | 4.9 | 0.0 | 4.9 | 0.00175303 | 2.35575 |
| UNC84A | 5.0 | 0.0 | 5.0 | 0.937022 | -1.0124 |
| ZFP36L2 | 6.7 | -1.3 | 5.4 | 0.23572 | 1.29509 |
| SND1 | 5.5 | 0.0 | 5.5 | 0.0692574 | 1.1901 |
| RHOBTB3 | 5.7 | 0.0 | 5.7 | 0.000843237 | 3.72639 |
| UBAP2L | 5.9 | 0.0 | 5.9 | 0.480692 | 1.07667 |
| NIPA2 | 6.0 | 0.0 | 6.0 | 0.506712 | 1.11402 |
| SHOX2 | 7.2 | -1.0 | 6.2 | 0.00086326 | 2.60447 |
| SYNCRIP | 6.6 | 0.0 | 6.6 | 0.10055 | 1.09704 |
| PTPN4 | 7.0 | 0.0 | 7.0 | 0.0971159 | 1.20553 |
| BAHCC1 | 7.0 | 0.0 | 7.0 | 0.828085 | 1.01182 |
| RASD1 | 7.1 | 0.0 | 7.1 | 0.118994 | 1.29799 |
| SLC16A2 | 8.9 | 0.0 | 8.9 | 0.396463 | -1.20368 |

Table 4: List of predicted weighted target scores for the most significantly regulated miRNA in OSCC and the matching changes in gene expressions. 50 genes with the highest and lowest combined inhibitory score are shown.

| Associated Network | Functions Score |
| --- | --- |
| 1 Connective Tissue Disorders, Genetic Disorder, Ophthalmic Disease | 49 |
| 2 Connective Tissue Disorders, Genetic Disorder, Dermatological Diseases and Conditions | 41 |
| 3 Gene Expression, Cellular Movement, Embryonic Development | 33 |
| 4 Cell-To-Cell Signaling and Interaction, Cellular Assembly and Organization, Developmental Disorder | 33 |
| 5 Carbohydrate Metabolism, Small Molecule Biochemistry, Connective Tissue Development and Function | 32 |

Table 5: IPA Core Analysis: Top associated network functions score of significant differentially expressed genes in OSCC versus oral cavity controls

| Diseases and Disorders | | |
| --- | --- | --- |
| Name | p-value | Number of genes |
| Connective Tissue Disorders | 7,37E-16 - 1,04E-03 | 144 |
| Genetic Disorder | 7,37E-16 - 1,06E-03 | 427 |
| Cancer | 1,50E-12 - 1,36E-03 | 249 |
| Cardiovascular Disease | 1,52E-11 - 1,25E-03 | 189 |
| Organismal Injury and Abnormalities | 1,47E-09 - 1,41E-03 | 68 |

Table 6: IPA Core Analysis: Predicted diseases and disorders based upon significant differentially expressed genes in Oral SCC versus Oral Controls

| Molecular and Cellular Functions | | |
| --- | --- | --- |
| Name | p-value | Number of genes |
| Cellular Movement | 2,99E-16 - 1,41E-03 | 151 |
| Cell Morphology | 9,60E-15 - 1,38E-03 | 116 |
| Cell-To-Cell Signaling and Interaction | 1,23E-12 - 1,36E-03 | 122 |
| Cellular Development | 1,12E-11 - 1,38E-03 | 194 |
| Cellular Growth and Proliferation | 2,55E-08 - 1,29E-03 | 213 |

Table 7: IPA Core Analysis: Molecular and cellular functions based upon significant differentially expressed genes in OSCC versus oral cavity controls

| Physiological System Development and Function | | |
| --- | --- | --- |
| Name | p-value | Number of genes |
| Tissue Development | 1,19E-15 - 1,28E-03 | 178 |
| Cardiovascular System Development and Function | 8,89E-14 - 1,41E-03 | 105 |
| Organismal Development | 8,89E-14 - 9,50E-04 | 119 |
| Skeletal and Muscular System Development and Function | 1,06E-10 - 1,28E-03 | 95 |
| Organismal Survival | 5,27E-07 - 8,98E-07 | 79 |

Table 8: IPA Core Analysis: Physiological system development and function based upon significant differentially expressed genes in OSCC versus oral cavity controls

| Top Canonical Pathways | | |
| --- | --- | --- |
| Name | p-value | Ratio (number of genes used in the pathway) |
| Agrin Interactions at Neuromuscular Junction | 8,63E-07 | 14/69 (0,203) |
| TGF-b Signaling | 2,09E-05 | 13/83 (0,157) |
| Axonal Guidance Signaling | 2,34E-05 | 34/410 (0,083) |
| Hepatic Fibrosis / Hepatic Stellate Cell Activation | 2,93E-05 | 18/147 (0,122) |
| Neuregulin Signaling | 3,02E-05 | 14/105 (0,133) |

Table 9: IPA Core Analysis: Top canonical pathways based upon significant differentially expressed genes in OSCC versus oral cavity controls

| Top Up-regulated and down-regulated molecules | |
| --- | --- |
| Molecules | Foldchange |
| POSTN | 86,707 |
| MMP1 (includes EG:4312) | 83,006 |
| MMP3 | 28,165 |
| PTHLH | 20,019 |
| COL12A1 | 18,061 |
| MMP10 | 16,907 |
| SERPINE1 | 16,072 |
| LAMC2 | 15,894 |
| SPP1 | 15,274 |
| TNC | 15,136 |
| KRT4 | -27,390 |
| FLG (includes EG:2312) | -18,549 |
| CEACAM7 | -18,132 |
| CRNN | -17,872 |
| CLDN17 | -16,491 |
| IL1F6 | -15,234 |
| MAL | -14,377 |
| CLCA4 (includes EG:22802) | -13,200 |
| C21ORF81 | -11,127 |
| FMO2 | -9,948 |

Table 10: IPA Core Ananlysis: Top Up-regulated and down-regulated genes in OSCC versus oral cavity controls

| **Network** | Score |
| --- | --- |
| 1 RNA Post-Transcriptional Modification, Cancer, Gene Expression | 42 |
| 2 Gene Expression, Developmental Disorder, Neurological Disease | 40 |
| 3 Cellular Assembly and Organization, Cell Morphology, Cellular Compromise | 38 |
| 4 Cancer, Carbohydrate Metabolism, Lipid Metabolism | 36 |
| 5 Gene Expression, Cell Signaling, Cancer | 32 |

Table 11:IPA Core Analysis: Top associated network functions score based upon predicted miRNA target genes (452 genes, weighted inhibitory score >2+/-) in OSCC

| **Top Bio Functions** | | |
| --- | --- | --- |
| **Name** | **p-value** | **Number of genes** |
| Cancer | 1,32E-06 - 1,84E-02 | 153 |
| Developmental Disorder | 1,46E-05 - 1,45E-02 | 66 |
| Infection Mechanism | 1,14E-04 - 8,79E-03 | 67 |
| Neurological Disease | 1,85E-04 - 3,85E-03 | 203 |
| Reproductive System Disease | 2,75E-04 - 1,84E-02 | 53 |

Table 12: IPA Core Analysis: Predicted diseases and disorders based upon predicted miRNA target genes (452 genes, weighted inhibitory score >2+/-) in OSCC

| **Molecular and Cellular Functions** | | |
| --- | --- | --- |
| **Name** | **p-value** | **Number of genes** |
| Gene Expression | 2,53E-18 - 1,79E-02 | 180 |
| Cellular Growth and Proliferation | 1,42E-07 - 1,83E-02 | 176 |
| Post-Translational Modification | 2,71E-07 - 1,69E-02 | 86 |
| Cell Death | 5,61E-07 - 1,79E-02 | 170 |
| Cell Cycle | 3,44E-06 - 1,79E-02 | 87 |

Table 13:IPA Core Analysis: Molecular and cellular functions based upon predicted miRNA target genes (452 genes, weighted inhibitory score >2+/-) in OSCC.

| **Physiological System Development and Function** | | |
| --- | --- | --- |
| **Name** | **p-value** | **Number of genes** |
| Organismal Development | 3,29E-12 - 1,55E-02 | 104 |
| Embryonic Development | 6,45E-07 - 1,76E-02 | 81 |
| Tumor Morphology | 8,85E-07 - 1,83E-02 | 34 |
| Tissue Development | 6,92E-06 - 1,79E-02 | 93 |
| Organismal Survival | 1,22E-05 - 4,82E-03 | 72 |

Table 14: IPA Core Analysis: Physiological system development and function based upon predicted miRNA target genes (452 genes, weighted inhibitory score >2+/-) in OSCC.

| **Top Canonical pathways** | | |
| --- | --- | --- |
| **Name** | **p-value** | **Ratio** (number of genes used in the pathway) |
| mTOR Signaling | 6,18E-08 | 21/164 (0,128) |
| HGF Signaling | 1,22E-07 | 17/107 (0,159) |
| IGF-1 Signaling | 1,8E-07 | 16/100 (0,16) |
| ERK/MAPK Signaling | 2,33E-07 | 23/204 (0,113) |
| PDGF Signaling | 1,39E-06 | 13/79 (0,165) |

Table 15: IPA Core Analysis: Top canonical pathways based upon predicted miRNA target genes (452 genes, weighted inhibitory score >2+/-) in OSCC

**Top up-regulated and down-regulated molecules**

| **Molecules** |  | **Combined weighted inhibitory score** |
| --- | --- | --- |
| SLC16A2 |  | 8,924 |
| RASD1 |  | 7,102 |
| BAHCC1 |  | 7,027 |
| PTPN4 |  | 6,989 |
| SYNCRIP |  | 6,613 |
| SHOX2 |  | 6,202 |
| UBAP2L |  | 5,851 |
| RHOBTB3 |  | 5,727 |
| SND1 |  | 5,525 |
| ZFP36L2 |  | 5,408 |
| PIK3C2A |  | -7,244 |
| KANK1 |  | -5,735 |
| JAZF1 |  | -5,692 |
| NUMB |  | -5,480 |
| SLC2A3 |  | -5,421 |
| TRAF6 |  | -5,342 |
| ZFP36L1 |  | -5,112 |
| TIMP3* |  | -5,098 |
| GATAD2B |  | -5,079 |
| YLPM1 |  | -4,991 |

Table 16: IPA Core Analysis: Top up- and down-regulated predicted miRNA target genes used for the analyses.
